# Core-Level Spectroscopy of 2-Thiouracil at the Sulfur L_1_- and L_2,3_-Edges Utilizing a SASE Free-Electron Laser

**DOI:** 10.3390/molecules26216469

**Published:** 2021-10-26

**Authors:** Fabiano Lever, Dennis Mayer, Jan Metje, Skirmantas Alisauskas, Francesca Calegari, Stefan Düsterer, Raimund Feifel, Mario Niebuhr, Bastian Manschwetus, Marion Kuhlmann, Tommaso Mazza, Matthew Scott Robinson, Richard J. Squibb, Andrea Trabattoni, Måns Wallner, Thomas J. A. Wolf, Markus Gühr

**Affiliations:** 1 Institut für Physik und Astronomie, Universität Potsdam, 14476 Potsdam, Germany; fabiano.lever@uni-potsdam.de (F.L.); dmayer@uni-potsdam.de (D.M.); metje.jan@gmail.com (J.M.); mniebuhr@uni-potsdam.de (M.N.); matthew.robinson@desy.de (M.S.R.); 2 Deutsches Elektronen Synchrotron (DESY), 22607 Hamburg, Germany; skirmantas.alisauskas@desy.de (S.A.); stefan.duesterer@desy.de (S.D.); bastian.manschwetus@desy.de (B.M.); marion.kuhlmann@desy.de (M.K.); 3 Center for Free-Electron Laser Science (CFEL), Deutsches Elektronen Synchrotron (DESY), Notkestraße 85, 22607 Hamburg, Germany; francesca.calegari@desy.de (F.C.); andrea.trabattoni@desy.de (A.T.); 4 The Hamburg Centre for Ultrafast Imaging, Universität Hamburg, 22761 Hamburg, Germany; 5 Institut für Experimentalphysik, Universität Hamburg, 22761 Hamburg, Germany; 6 Department of Physics, Gothenburg University, SE-41296 Gothenburg, Sweden; raimund.feifel@physics.gu.se (R.F.); richard.squibb@physics.gu.se (R.J.S.); mans.wallner@physics.gu.se (M.W.); 7 European XFEL, 22869 Schenefeld, Germany; tommaso.mazza@xfel.eu; 8 Stanford PULSE Institute, SLAC National Accelerator Laboratory, Menlo Park, CA 94025, USA; thomas.wolf@stanford.edu

**Keywords:** X-ray, photoelectron, sulfur, thiouracil, nucleobases, Coster–Kronig, Auger–Meitner, NEXAFS, FLASH

## Abstract

In this paper, we report X-ray absorption and core-level electron spectra of the nucleobase derivative 2-thiouracil at the sulfur L_1_- and L_2,3_-edges. We used soft X-rays from the free-electron laser FLASH2 for the excitation of isolated molecules and dispersed the outgoing electrons with a magnetic bottle spectrometer. We identified photoelectrons from the 2p core orbital, accompanied by an electron correlation satellite, as well as resonant and non-resonant Coster–Kronig and Auger–Meitner emission at the L_1_- and L_2,3_-edges, respectively. We used the electron yield to construct X-ray absorption spectra at the two edges. The experimental data obtained are put in the context of the literature currently available on sulfur core-level and 2-thiouracil spectroscopy.

## 1. Introduction

Recent years have seen increasing interest in the study of sulfur-substituted nucleobases, known as thionucleobases, for applications in medicine and biochemistry [1,2]. They differ from their canonical counterparts in their response to UV radiation. The substitution of an oxygen atom with the much heavier sulfur atom significantly changes the potential energy landscape, affecting how the molecules interact with light. The absorption spectrum is shifted from UVC into the UVA range, and the resulting excitation produces long-lived triplet states [3,4,5,6,7,8]. Their reactive triplet state makes thionucleobases useful as cross-linking agents [9,10], as well as candidates for photoinduced cancer treatment [11,12]. 

Ultrafast radiationless transitions are crucial in funneling the molecular population from the initially excited ^1^ππ* states into the long-lived ^3^ππ* states. The details of these dynamics have been the topic of theoretical and experimental efforts (see Ref. [8] and the references therein). The particular thionucleobase 2-thiouracil (2-tUra) is among the most studied systems. Its static potential energy landscape properties indicate the existence of several conical intersection (CI) seams [13]. Dynamical simulations predict how an initially UV-excited population traverses the CI regions to end up in the lowest ^3^ππ* state. Experiments using UV pump and visible-ultraviolet probe pulses in solvents [4,14] and in the gas phase [14,15,16] confirm the ultrafast nature of the molecular transitions. The combination of dynamics predictions with experimental transient spectra present a powerful approach to deduce molecular dynamics. These combined studies suggested ultrafast sub-picosecond transitions from the ^1^ππ* to a ^1^nπ* ‘doorway’ state, from which the lower-lying triplet states are accessed [14,16].

Using ultrafast X-ray pulses to probe molecular dynamics via core-electron excitations provides insight into the molecular dynamics, a process which is complementary to the well-established probe methods that utilize valence electron transitions [17]. X-rays provide an element-sensitive probe as the core-level electron binding energies differ strongly among elements. The tight binding afforded also makes this method highly spatially selective. This particular advantage has been used to examine the ^1^nπ* channel via UV-pump X-ray probe studies on thymine [18,19]. For 2-tUra, we used the sulfur L-edges to probe the local dynamics at the C-S bond using time-resolved Auger–Meitner spectroscopy [20]. In addition, we could attribute electronic states using the excited-state chemical shift (ESCS) resulting from the local charge at the sulfur atom probed in time-resolved X-ray photoelectron studies [21].

In this paper, we present a static X-ray spectroscopic study of 2-tUra performed at the sulfur L-edge. Our work includes near-edge absorption fine structure (NEXAFS) spectra at the L_1_- and L_2,3_-edges, as well as photoelectron spectra involving the 2s and 2p core-holes. We furthermore investigate the Auger–Meitner spectra induced by sulfur 2p vacancies and the Coster–Kronig decay of the sulfur 2s core-hole as a function of X-ray energy. 

## 2. Results

**Figure 1:**
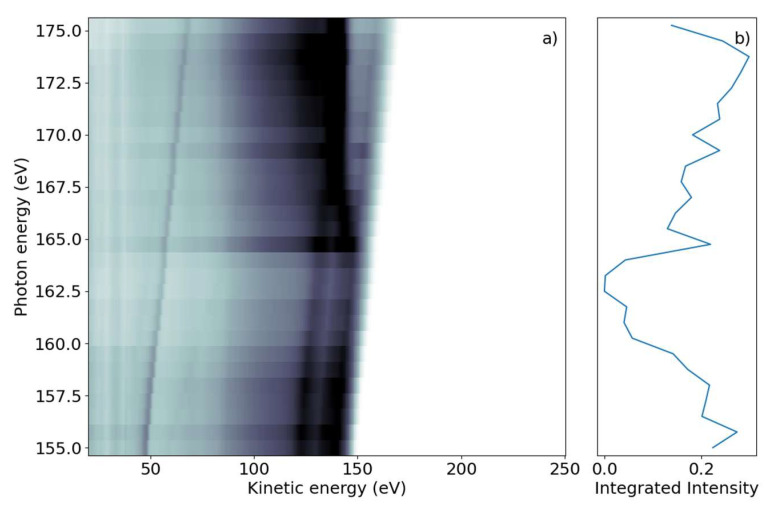
(**a**) Photon energy vs. electron kinetic energy for the 2-tUra sulfur 2p edge. The valence emission can be discerned as dispersing diagonal lines. Resonant and non-resonant Auger–Meitner emission splits off from the valence signal when the photon energy reaches the 2p binding energy. (**b**) NEXAFS spectrum obtained by integrating the electron emission intensity over the whole kinetic energy range. The 2p edge marks an increase in emitted signal, with some spectral features visible as peaks in the NEXAFS.

We first investigated the photon-energy range of the sulfur L_2,3_-edge creating a sulfur 2p core hole through either resonant transitions to core-valence excited states or non-resonant promotion of the core electron into the continuum. The FEL photon energy hυ was scanned over the ionization edge region from hυ = 155 to 175 eV, with 0.75 eV steps in randomized order; the averaged FEL bandwidth was about 4 eV. For each photon energy setting, a full electron spectrum of 2-tUra was recorded. The electron time-of-flight spectra were converted to the kinetic energy scale by taking the Jacobian correction in the binning process into account. The results are shown in Figure 1a, with electron spectral intensity shown in the form of grayscale false-color code as a function of electron kinetic energy and photon energy. 

The 2D spectrum shows two qualitatively different groups of features. First, we discerned a group of maxima that did not change their kinetic energy as the photon energy was varied. We refer to these as ‘non-dispersing’ lines or bands. The strongest is a broad line at 140 eV with a width of ~9 eV, which appears from photon energies of 167 eV and higher. This component is accompanied by a weaker one at E_kin_ = 130 eV and a smaller background towards lower kinetic energies.

The second group of features are ‘dispersing’, meaning they change their kinetic energy with hυ. In the photon energy range of 155–163 eV, we observed several such lines which changed E_kin_ linearly with hυ in the kinetic energy range above 120 eV. The highest kinetic energy lines also continued to be visible for larger photon energies up to 175 eV. A further dispersing line starting at E_kin_ = 48 eV at hυ = 155 eV also linearly changed its kinetic energy with hυ. This line is not part of the molecule and we discuss it further in the discussion below. An additional transition region was visible around hυ = 165 eV to 167 eV, where dispersive features convert into non-dispersive bands. In this region, a line with ‘negative’ dispersion seems to change from high to lower kinetic energies around E_kin_ = 140 eV. We integrated the full two-dimensional spectrum over the kinetic energy range and present it as a function of photon energy in Figure 1b. The NEXAFS spectrum obtained in this way shows a decreasing electron yield (absorption) from the lowest hυ to about 165 eV, where the absorption rises. This rise occurs over a photon energy interval of ~2 eV. 

**Figure 2:**
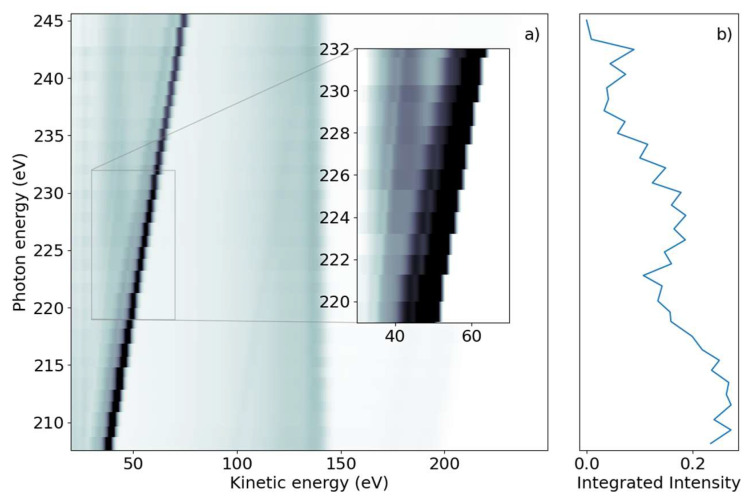
(**a**) Photon energy vs. kinetic energy for the 2-tUra sulfur 2s edge, with the photon energy varying from 208 eV to 245 eV. The bright diagonal feature is the dispersing 2p photoelectron line. A satellite photoelectron line is visible to the left of the main feature. Non-resonant 2p Auger–Meitner electron emission can be seen in the 100 eV to 150 eV range. Coster–Kronig electrons from the 2p -> 2s decay are visible at 40 eV for photon energies above 220 eV. The dispersing dip overlapping the Coster–Kronig feature visible in the inset is an artifact of the readout electronics. (**b**) NEXAFS spectrum obtained by integrating the emission intensity over the whole kinetic energy range.

We subsequently demonstrate the energy range around the sulfur L_1_-edge, which creates features connected to a sulfur 2s core-hole. We scanned the photon energy in the range of 206 eV to 240 eV, with 1 eV steps. Figure 2a shows a false-color 2D spectrum of electron yield as a function of kinetic energy and photon energy. Similar to the L_2,3_ spectrum, we again identified dispersing and non-dispersing lines. The most prominent dispersing feature changes E_kin_ linearly with hυ from E_kin_ = 38.5 eV to 75 eV over the full range shown in Figure 2a. The line is accompanied by a weaker dispersing line shifted by 6 eV towards lower kinetic energy. In addition, weaker dispersing features from valence ionization are visible for kinetic energies over 150 eV and up to 200 eV for the lowest photon energy. 

In the lower kinetic energy range, we observed a non-dispersing broad line centered around E_kin_ = 42.5 eV. At higher kinetic energies, we observed a non-dispersing band around E_kin_ = 140 eV with a tail towards lower energies. Analogous to the L_2,3_-edge, we generated a NEXAFS spectrum from the integrated electron yield, shown in Figure 2b. In addition to the decrease in intensity from lower to higher photon energies, an absorption increase starting at hυ = 222 eV with a maximum at hυ = 227 eV was observed.

## 3. Discussion

We first discuss the spectra at the sulfur L_2,3_-edge. According to calculations [22], the ionization potential of sulfur is given as 162.5 eV and 163.6 eV for the two spin-orbit split components 2p_3/2_ and 2p_1/2_, respectively. Photoelectron measurements of 2-tUra found the ionization potential values to be 168.17 eV and 169.37 eV for the spin-orbit split components [23]. The photon energy window from hυ = 155 eV to 176 eV in Figure 1 thus spans from well below to above the ionization potential.

**Figure 3:**
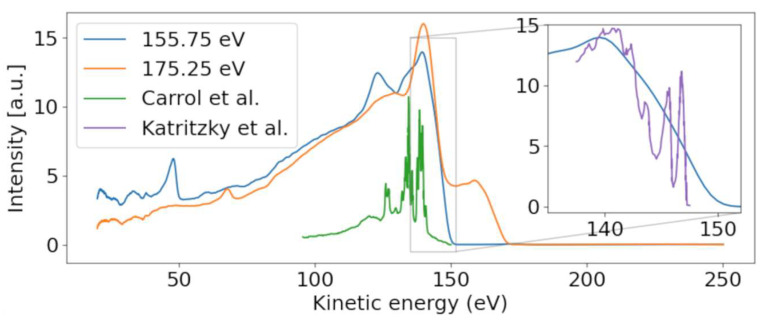
Photoelectron spectra for photon energies above (175.25 eV) and below (157.25 eV) the sulfur 2p binding energy. A dispersive behavior of the valence band is visible, moving from ~140 to ~160 eV with increasing photon energy. Our valence spectrum at hυ = 155.75 eV is compared with a He-lamp-induced valence photoelectron spectrum (purple) from Ref. [24] in the inset, which is scaled in kinetic energy according to the difference of photon energies used in the experiments. Conversely, the Auger–Meitner feature is only present for the higher photon energy. The Auger–Meitner data are compared with the sulfur Auger–Meitner spectrum of OCS (green) from Ref. [26].

At the lowest photon energies, the spectrum must be dominated by valence emission, and we can clearly identify dispersing features with a high energy edge around 150 eV kinetic energy. We thus compare the electron spectrum to the He-lamp induced valence photoemission spectrum taken over a range of only 10 eV (from 8 to 18 eV binding energy) [24]. Figure 3 shows a photoelectron spectrum taken at FLASH2 at hυ = 155.75 eV (blue line). The inset of Figure 3 compares a small region of that spectrum with the photoelectron spectrum obtained using the He (I) line at hυ = 21.2 eV. While the He spectrum shows rich detail attributed to photoemission from different valence orbitals [24], our spectrum at FLASH2 is only weakly modulated as a function of E_kin_. The ionization potential overlaps with the measured ionization potential of 8.8 eV [24]. The poor modulation of the FLASH2 valence photoelectron spectrum in Figure 1 and Figure 3 is a combined effect of the photon energy bandwidth of 4 eV and the reduced resolution of the magnetic bottle spectrometer at these comparatively high kinetic energies. The magnetic bottle was operated at retardation of only 5 eV; taking the measured 1/40 resolution [25], we arrive at a feature width of about 4 eV at E_kin_ = 150 eV. The valence features disperse with a slope of one in hυ per eV in E_kin_ throughout the whole measurement range, confirming the use of fundamental undulator radiation.

A second dispersive feature, starting at E_kin_ = 47.9 eV, has a binding energy of 108 eV (see also Figure 2). The slope, equal to the slope of the valence lines, indicates its origin from photoemission with the fundamental of the undulator. We suggest that this line stems from the Al tip of the oven, sitting at a distance of a few mm from the interaction region but still being hit by some halo of the X-ray beam. The 2s line of Al is nominally expected to be around 120 eV, but due to patch charges at the oxidized tip, this line might be shifted by a few eV towards its apparent binding energy of 108 eV.

We now concentrate on the non-dispersive features. At the limit of the highest photon energy in Figure 1, we observe a non-dispersive band peaking at 140 eV, shown in the orange line in Figure 3. As the photon energy of 175.25 eV is above the ionization limit for the sulfur 2p electrons, we assume that these features belong to non-resonant Auger–Meitner (NAM) decay of 2-tUra. An Auger–Meitner spectrum of the molecule is not available; we therefore compare the features with a sulfur L-MM Auger–Meitner spectrum of OCS (green line Figure 3) from Ref. [26]. The OCS reference shows four dominant groups of lines, which are attributed to different bands of dicationic final states in Ref. [26]. Generally, we observe less resolved features than in the OCS reference. On the one hand, this is due to the reduced resolution of our magnetic bottle spectrometer. On the other hand, larger species such as 2-tUra tend to not show resolved features on the eV scale. This argument can be made in analogy to thymine and its fragment isocyanic acid (HNCO). While the Auger–Meitner spectrum of the latter shows details on an eV scale in analogy to OCS, thymine only exhibits broad bands about 10 eV wide [27]. This effect is due to the increased density of final dicationic states in NAM decay for growing molecular size. The different bands at 140 and 130 eV correspond to broadened bands in the OCS spectrum. We do not know the exact electronic configuration of the valence dicationic states after Auger decay of the sulfur core-hole. However, the sulfur atom has two valence orbitals that must be strongly involved in the sulfur core hole decay: a strongly bound 3s and a shallow bound 3p valence orbital. A decay involving strongly bound valence orbitals in dicationic states leads to less kinetic energy of the Auger electron. Therefore, a very coarse interpretation can attribute the different band ‘humps’ at 140, 130, and 105 eV to dicationic decays with two valence holes dominated by sulfur 3p^−2^, 3p^−1^3s^−1^, and 3s^−2^ configurations, respectively. The hole-character refers to the sulfur atomic orbital contained in the molecular orbitals. 

The NEXAFS spectrum in Figure 1b shows an intensity decrease from lower hυ up to about 164 eV, where the integrated electron yield in the spectrum increases. This is about 4 eV below the first sulfur 2p binding energy and thus in the region of core-to-valence resonances from 2p core levels to unoccupied valence levels. The valence state in the transition needs to fulfill symmetry requirements, meaning it needs to contain either atomic sulfur s or d orbitals. As we do not observe any fine structure because of the comparatively large bandwidth, we have not pursued any calculations of the unoccupied valence electronic states. The lowest unoccupied states of 2-tUra, the so-called π* resonances, are dominated by sulfur atomic character. As in every NEXAFS spectrum, a dense series of Rydberg states with many different atomic contributions spans from the lowest resonances up to the ionization limit. There is currently no NEXAFS reference data available for 2-tUra. We thus compare our data to NEXAFS spectra of OCS and CS_2_ at the sulfur L_2,3_-edge [28], as well as on dimethyl disulfide at the sulfur L_2,3_-and also L_1_-edges [29]. In CS_2_ as well as OCS, the first resonances are described by 2p_3/2_ and 2p_1/2_ to π* transitions, located around 163–164 eV and 164–165 eV for CS_2_ and OCS, respectively. Higher resonances are attributed to Rydberg-transitions with 4s and 3d sulfur character. Above the ionization limit, the spectra of CS_2_ and OCS show a broad shape resonance.

The kinetic energy-resolved region of the core-valence resonances shows so-called resonant Auger–Meitner processes. The resonant Auger–Meitner (RAM) decay [30] has been studied in several molecules, from diatomic to quadratomic [31,32]. Part of our group has studied RAM decay in the nucleobase thymine, where it has been used to infer molecular excited state dynamics [33]. In RAM decay, the initial core-excited neutral state decays into a cationic state, in contrast to decay to dicationic states, as is the case for core-ionized states in a NAM process. We clearly see the effect of the final state in the transition of the RAM to the NAM decay of 2-tUra. At the 1s-π* resonance, the most prominent feature in the RAM sits at 146 eV, which then transforms into the NAM feature at 140 eV kinetic energy. The shift results from a more attractive dicationic potential for the outgoing Auger–Meitner electron in NAM as compared with the cationic potential in RAM. The transition region of RAM to NAM in thymine shows a very similar shift, appearing as dispersion in the wrong direction, i.e., the kinetic energy decreases as the photon energy increases (see Figure 2 in Ref. [33]). The spectrum in Figure 1 does not possess sufficient kinetic energy resolution to distinguish participator (final states are identical to outer valence ionized final states) and spectator decay (final states possess an excited valence electron corresponding to inner valence ionized final states), in contrast to Ref. [33].

We now discuss features at the L_1_-edge, which implies a sulfur 2s hole being created upon X-ray interaction. Unlike the L_2,3_-edges, only one core-hole state is created here due to the absence of spin-orbit coupling. According to calculations, the binding energy of the sulfur 2s-ionized state is 230 eV [22]. The photon energy window in Figure 2, therefore, contains the sulfur L_1_-edge. 

**Figure 4:**
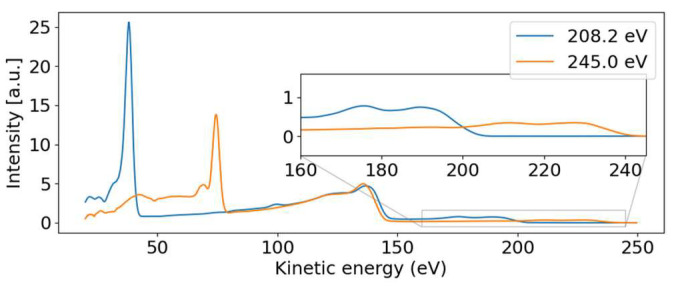
Photoelectron spectra for photon energies above (~245 eV) and below (~208 eV) the 2s binding energy. The dispersive behavior of the 2p photoelectron line is visible, moving from 39.8 to 74.4 eV with increasing photon energy. The 2p non-resonant Auger–Meitner band from 100 eV to 144 eV remains stable and is independent of the photon energy. The dispersive valence features are visible in the inset.

For the discussion, we first concentrate on the most prominent feature in Figure 2: the dispersive line presented more prominently in the inset. Figure 4 presents some lineouts of kinetic energy spectra at specific photon energies. We find a kinetic energy of 39.8 eV at the photon energy of 209.35 eV, corresponding to a binding energy of 169.5 eV. The linewidth is about 4 eV, masking any splitting below that width. We thus conclude that the dispersive feature must be the sulfur 2p photoelectron line at the binding energies of 168.17 eV and 169.37 eV for the 2p_3/2_ and 2p_1/2_ spin-orbit split components, respectively [23]. The experimentally found dispersion fits to ionization by the first order of undulator radiation from FLASH2. 

Parallel to the main photoelectron line, a correlation satellite line [34] appears 5 eV below in kinetic energy, most clearly visible in the hυ = 240 eV spectrum (orange line in Figure 4) at a kinetic energy of 68 eV. In an orbital picture, the structure corresponds to a shake-up process from an occupied to an unoccupied valence orbital induced by the sulfur 2p photoionization. Thus, the photoelectron has the energy of the main line reduced by the energy to accomplish that shake-up. Satellite structures of the sulfur 2p photoelectron line have also been documented for comparatively small molecules such as SF_6_ [35] or sulfur atoms on a metal surface [36]. For the latter, a similar shift is observed. 

The non-dispersive part is composed of the sulfur 2p NAM decay in the 100 to 150 eV kinetic energy range. For photon energies above the 2s binding energy, we also see a non-dispersive feature with a kinetic energy of about 40 eV. We attribute this second feature to the Coster–Kronig spectrum of the 2s hole, which is dominated by one broad peak at 42.5 eV kinetic energy, visible in the orange line of Figure 4. Photon energies above the 2s binding energy are able to create a 2s core hole, which is rapidly filled by a 2p electron in the form of Coster–Kronig decay, ejecting a valence electron in the process. The observed kinetic energy is therefore given by the 2s-2p energy gap, minus the valence binding energy, and is independent of the photon energy that produced the 2s core hole. To our knowledge, there are no S 2s Coster–Kronig spectra documented for molecules. We thus compare the spectra to those of sulfur atoms on surfaces from Ref. [36], showing two bands at 40 and 50 eV kinetic energy, which falls energetically within our observed broad band. Those bands are attributed to 2s^−1^ Coster–Kronig decays with a 2p^−1^ 3s^−1^ and 2p^−1^ 3p^−1^ final state accompanied by a charge transfer from the sulfur atom into the substrate. 

The 2p induced NAM decay is visible in both the blue and orange lines of Figure 4, where a slight shift in the peak position is observed. At low photon energies, the 2p decay is induced by 2p photoelectron emission. For the higher photon energies, however, the 2p NAM decay can be induced by both 2p and 2s photoelectron emission. In case of 2s ionization, the 2s Coster–Kronig decay will create a valence hole and 2p-core hole; the latter will then decay via NAM channels. We suggest that the Auger spectra of NAM and Coster–Kronig induced NAM are different because of the additional valence hole. Thus, the shift is caused by photon energy-dependent changes in the relative intensities of the NAM decay channels originating from either 2s or 2p ionization. 

For the L_1_-NEXAFS spectrum, we observed the peak of the absorption feature as being between hυ = 225 and 230 eV. The ‘generic’ binding energy of the 2s electron of 230 eV likely needs to be shifted upwards in the molecule by a few eV in analogy to the 2p electron. Thus, we end up with 2s-π* transitions at the maximum of the NEXAFS spectrum. Again, because of a lack of molecular NEXAFS spectra, we point to Ref. [36] for comparison, where the maximum of the 2s-3p_z_ absorption was observed at 225 eV.

The relatively large bandwidth of the X-rays—of up to 2%—limits the energy resolution in NEXAFS and resonant Auger–Meitner and photoelectron spectroscopy. Nevertheless, we were able to discern features that are attributed to core-valence resonances and core-level electron binding energies. The non-resonant Auger–Meitner and Coster–Kronig features are independent of the initial photon energy and bandwidth. Their shape is therefore governed by the resolution of the electron spectrometer and the number of electronic states accessible by Auger–Meitner and Coster–Kronig decay in large molecules. 

## 4. Materials and Methods

The data were obtained at the FL24 beamline of the FLASH2 free-electron laser (FEL) [37,38] as part of a more extensive investigation of the dynamics following UV excitation [20,21]. An instrument containing sample injection and a magnetic bottle-type electron spectrometer (URSA-PQ, German: Ultraschnelle Röntgenspektroskopie zur Abfrage der Photoenergiekonversion in Quantensystemen, English: Ultrafast X-ray spectroscopy for probing photoenergy conversion in quantum systems)—designed and built by the group at University of Potsdam—was connected to the beamline [25].

At the FLASH FEL, the X-rays are produced by self-amplified spontaneous emission (SASE), and we used photon energies at the FLASH2 branch in the range of 150 eV to 250 eV tuned by variable gap undulators. Rare-gas photoelectron spectrometers built in the beamline, the OPIS (Online PhotoIonization Spectrometer) [39], were used to determine the wavelength and spectrum of the emitted light; for the machine settings used in this study, the average bandwidth (including jitter) was found to be 4 eV. This kind of spectrometer does not provide shot-to-shot resolved data. We instead averaged the OPIS spectra for a complete run with minutes of data to determine the average photon energy value for each photon energy bin. The different step sizes for the two energy scans (1 eV and 0.75 eV) were chosen to allow for efficient use of experimental time; a finer step size of about 4 eV effective bandwidth would not improve the data. We scanned with a slightly larger step size at the L_1_-edge, as we expected wider features at the L_1_-edge compared with the L_2,3_-edge.

The FEL delivers the radiation in trains of pulses with a repetition rate of 10 Hz [40]. Each train consists of 50 pulses at 200 kHz internal repetition rate, with an estimated pulse duration of 150 fs [25]. Pulse energy scans were carried out to avoid X-ray saturation of the obtained electron spectra. During the data collection, the average pulse energy used was 5 μJ. The X-rays are focused by means of Kirkpatrick–Baez (KB) mirrors to a spot of ~100 μm size, located in the interaction region of our magnetic bottle electron spectrometer (MBES) [41]. The X-rays are linearly polarized parallel to the axis of the spectrometer. 

**Figure 5:**
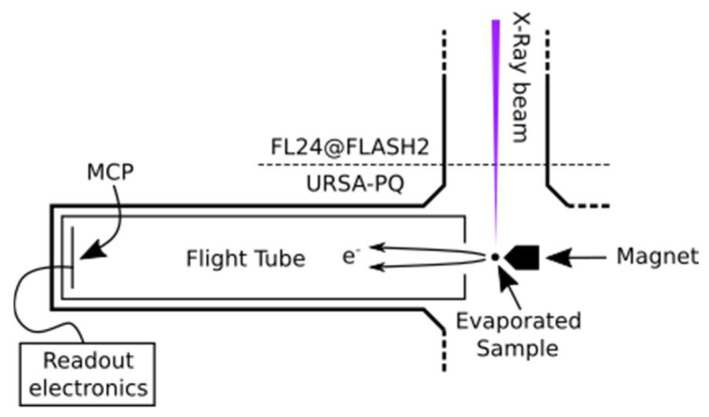
Sketch of the experimental setup. The soft X-rays from FLASH2 are focused into the interaction region of the magnetic bottle spectrometer, where the sample is provided by means of a capillary oven (located above the spectrometer, out of plane of the diagram). The emitted photoelectrons enter the flight tube and are detected by an MCP detector.

Figure 5 presents a sketch of the URSA-PQ apparatus. The use of an MBES allows for high collection efficiency. A permanent magnet with a soft iron pole produces a high magnetic field in the interaction region, which adiabatically changes into a homogeneous solenoid field that guides the confined electrons through an almost 2 m long flight tube [41]. The emitted electrons are detected by a multi-channel plate (MCP) assembly at the end of the flight tube. Their kinetic energy can then be obtained from the time-of-flight measurements. A time-of-flight spectrum was recorded for each pulse of the FEL, and the results were subsequently averaged. An electrostatic lens was used to apply a retardation potential to the electrons as they enter the flight tube, slowing them down and thus increasing the time-of-flight resolution. Calibration of the spectrometer was carried out using Kr-MNN Auger–Meitner electrons [25]. Using the calibration data together with a geometric model of the flight tube, a time-of-flight to kinetic energy conversion function was developed. This function was then adapted to enable the conversion of data acquired under different retardation settings. Specifically, we used recorded krypton spectra to construct a model of the spectrometer and extrapolated it to other retardation settings/energy ranges. The resolution (E/ΔE) was found to be around 40 under the chosen settings [25]. For each X-ray pulse, FLASH provides a shot-to-shot measurement of the pulse energy (photon number) through the use of gas-monitor detectors (GMD). We used these data to normalize our electron spectra.

The 2-tUra sample (acquired from Sigma-Aldrich (St. Louis, MO, USA) and used without further processing) was delivered by a capillary oven [42] heated to 150°C and located near the tip of the permanent magnet of the spectrometer. At this oven temperature, the only tautomer is the oxo-form [23], as noted in a comparison of gas-phase 2-tUra experiments [15,23]. 

## 5. Conclusions

Here, we present the results of an X-ray sulfur core-level investigation of 2-thiouracil. The data obtained—primarily covering previously unobserved energy ranges—provide novel information about this molecule which is complementary to the currently available literature. 

The valence and 2p photoelectron features present a dispersive character, with the electron kinetic energy linearly following the changes in photon energy. However, the Auger–Meitner and Coster–Kronig decay channels are associated with constant kinetic energies and do not show dispersion.

The previously measured value for the sulfur 2p binding energy in 2-tUra of 168 eV [23] is consistent with our observations for both the 2p photoelectron line kinetic energy and the onset of the 2p Auger–Meitner features. Moreover, our NEXAFS spectra show a similar position of the sulfur-2p edge as the already available ion-yield NEXAFS spectra of related sulfur-containing compounds [28], although with a significantly lower resolution in the finer details of our spectrum. This is due to the large bandwidth of the FEL radiation used, acting to broaden the observed features. The future use of a monochromator would help to increase the energy resolution, as in [19]. It increases the shot-to-shot fluctuations when used with SASE sources, but this can be corrected for on a single-shot basis.

To our knowledge, this work is the first available NEXAFS spectrum of 2-tUra at the sulfur 2p and 2s edges. 

The observed 2p Auger–Meitner spectrum does not present any fine structure or discernable lines. In other cases, such as OCS, pronounced features in the sulfur Auger–Meitner spectra were observed [26]. We attribute the lack of fine structure to the relatively high number of atoms forming 2-tUra, leading to a high density of dicationic states and thus leading to spectral congestion [27].

Resonant and non-resonant Auger–Meitner emissions can be distinguished by their kinetic energy, with resonant electrons showing a shift of about 10 eV toward higher kinetic energies. Similar shifts are observed in other molecules [32], and are attributed to the spectating electron affecting the energy landscape in which the decay takes place.

At the sulfur L_1_-edge, we observe the dispersive 2p photoline as well as a broad non-dispersing Coster–Kronig band at around 40 eV. Comparisons to a core level spectroscopy study of sulfur atoms on a metal surface confirm our interpretation of the features in the absence of comparable molecular data for this spectral region. 

## Data Availability

Data is available upon request.

## Sample Availability

Samples of the compounds are available from Sigma-Aldrich.
